# Exploring effective strategies in promoting diversity, equity, and inclusivity in surgery: scoping review

**DOI:** 10.1093/bjsopen/zrag020

**Published:** 2026-04-13

**Authors:** Afroza Sharmin, Rawan Ahmed, Alexander W Phillips

**Affiliations:** Department of Surgery and Cancer, Imperial College London, London, UK; Bioscience Education Department, King’s College London, London, UK; Northern Oesophagogastric Unit, Royal Victoria Infirmary, Newcastle upon Tyne, UK; School of Medical Education, Newcastle University, Newcastle Upon Tyne, UK

**Keywords:** DEI, women, minority, under-represented, surgeons, surgery

## Abstract

**Background:**

Diversity, equity, and inclusion is vital for improving patient care, reducing health disparities, and driving innovation in surgery. Despite this, surgical doctors who are women or from minority groups remain under-represented in surgery. Barriers including limited exposure, entrenched stereotypes, systemic biases, and lack of diverse role models persist, whereas effective strategies remain underexplored. This review evaluates existing diversity, equity, and inclusion interventions that improve recruitment, retention, and promotion in surgery.

**Methods:**

Following PRISMA-ScR guidelines, PubMed, Embase, and the Cochrane Library were searched in November 2024 and updated in September 2025 to include all studies published up to that date. Eligible studies ranged from October 2010 to August 2025. Methodological quality was assessed using an adapted 2018 Critical Appraisal Skills Programme checklist. Data were synthesized thematically to identify recurring intervention patterns.

**Results:**

From 928 articles, 19 studies were included. Four overarching themes emerged: (1) pipeline programmes (workshops and scholarships) fostered early exposure, with some reporting > 70% participants from minority groups and doubled surgical entry of women; (2) holistic recruitment strategies overcoming rigid academic cut-offs increased interview offers to participants from minority groups (up to 8%) while maintaining academic standards, with some programmes reporting zero attrition; (3) mentorship from medical school to early career yielded 86% fellowship pursuit and > 50% faculty appointments among surgeons from minority groups; and (4) comprehensive institutional efforts, including diversity committees and faculty bias training, achieved up to 55% women and 33% people from minority groups among new hires. Mentorship, holistic reviews, career-stage scholarships, and institutional accountability emerged as impactful measures.

**Conclusion:**

This review underscores the importance of integrated diversity, equity, and inclusion strategies providing evidence-based pathways to address systemic inequities and build a more inclusive surgical workforce.

## Teaser

This scoping review highlights evidence-based diversity, equity, and inclusion interventions that improve recruitment, retention, and promotion in surgical careers. Effective strategies include pipeline programmes, holistic recruitment, mentorship, and institutional initiatives, each showing measurable success in increasing representation of women and people from minority groups in surgery. These findings are timely, given the current discussion around diversity, equity, and inclusion, and offer globally transferable strategies applicable to different healthcare models.

## Introduction

Diversity in all its forms is increasingly recognized as essential for excellence in science^1^. Diversity enhances patient outcomes through patient–physician concordance^2,3^ and cultural competence^4–7^. Female surgeons^8^ are associated with lower patient mortality rates whereas physicians from ethnic minority backgrounds are more likely to serve marginalized communities and address disparities^9,10^. Diverse teams also drive innovation with increased participation of minority groups in medical research^11–13^. Despite these well documented benefits and legislations like the Equality Act (2010)^14^ promoting diversity, equity, and inclusion (DEI), the progress in surgery has been slow. Women represent just 17% of UK surgical consultants^15^ and people from ethnic minority backgrounds comprise only 25%^16^. In the USA, only 3–5% of attending surgeons involved in academic surgery come from under-represented groups^17^. This reflects the ‘leaky pipeline’ phenomenon^18–20^ alongside entrenched stereotypes, rigid training structures, economic challenges^21–23^, and lack of diverse mentors^24–26^. The 2020 Kennedy Review^27,28^ exposed unconscious bias, institutional racism, and sexism as persistent hurdles, further highlighted by social media campaigns, such as the *#Ilooklikeasurgeon* movement^29^.

Whereas previous literature often proposed theoretical frameworks without assessing tangible outcomes, recent studies^30–32^have begun to evaluate specific interventions. This scoping review aims to evaluate these implemented strategies to improve DEI in surgery by identifying common themes, analysing both short- and long-term outcomes across different career stages and uncovering barriers to implementation. This will provide evidence-based recommendations to support recruitment, retention, and promotion in surgery. This review addresses the gap by systematically examining interventions with measurable outcomes to advance surgical workforce diversity.

## Methods

This scoping review followed the PRISMA-ScR guidelines^33^. A comprehensive search was conducted across PubMed, Embase, and the Cochrane Library using Boolean operators and medical subject heading terms to maximize sensitivity and specificity. Key search terms included *Surg AND (Diversity OR Equity OR Inclusivity OR DEI OR EDI OR Underrepresented) AND (Strateg* OR *Initiative*). A full search strategy is provided in the *Supplementary Material* Methods. Citation tracking of relevant articles was also undertaken.

Studies were included if they (1) focused on surgical specialties, (2) included medical students and/or doctors in surgery, (3) assessed measurable DEI interventions, (4) were peer-reviewed, and (5) were published in English. No restrictions were placed on the publication year or country of origin. Exclusion criteria included studies outside surgical fields, those involving high school students or healthcare professionals other than doctors, descriptive reports without intervention strategies, non-peer-reviewed publications, and non-English studies. Two reviewers independently screened titles, abstracts, and full texts using EndNote, resolving discrepancies by consensus. The search was conducted in November 2024 and updated on 25 September 2025. Data were systematically extracted into a structured article matrix, capturing key variables on an Excel spreadsheet: study title, author, publication year, institution or country, surgical specialty, study design, intervention strategy, measured outcomes, and findings. The timeframe of eligible studies ranged from October 2010 to August 2025. Methodological quality was assessed using the adapted 2018 Critical Appraisal Skills Programme (CASP) checklist^34^. Bias assessment was integrated into the evaluation to ensure the validity of findings. This adapted CASP checklist can be found in *Table S1*.

A thematic synthesis approach was employed to identify patterns across studies by coding findings and grouping them into descriptive themes that emerged during full-text review. These themes were developed inductively through iterative re-reading of selected articles, based on similarities in intervention delivery, targeted career stage, and reported outcomes.

Due to the substantial heterogeneity in intervention types, target populations, methodological approaches, and measured outcomes, meta-analysis was deemed inappropriate. For consistency, the following definitions were applied: diversity generally encompasses gender, gender identity, race, ethnicity, socioeconomic status, age, neurodiversity, physical ability, sexual orientation, and religious affiliation^35^. Although this review addresses diversity comprehensively, the literature search did not yield studies focused on strategies beyond gender, race, and ethnicity. Equity is the fair treatment based on individual needs^36^ and inclusivity emphasizes a sense of belonging by embracing diverse perspectives^37^. The term under-represented in medicine (UiM) or under-represented minority seen interchangeably in the literature refers to racial and ethnic groups under-represented in the medical profession and this review adopts UiM for consistency. An intervention was considered effective if it improved application, recruitment, progression, or leadership representation of women or UiM individuals in surgical specialities.

## Results

**Fig. 1:**
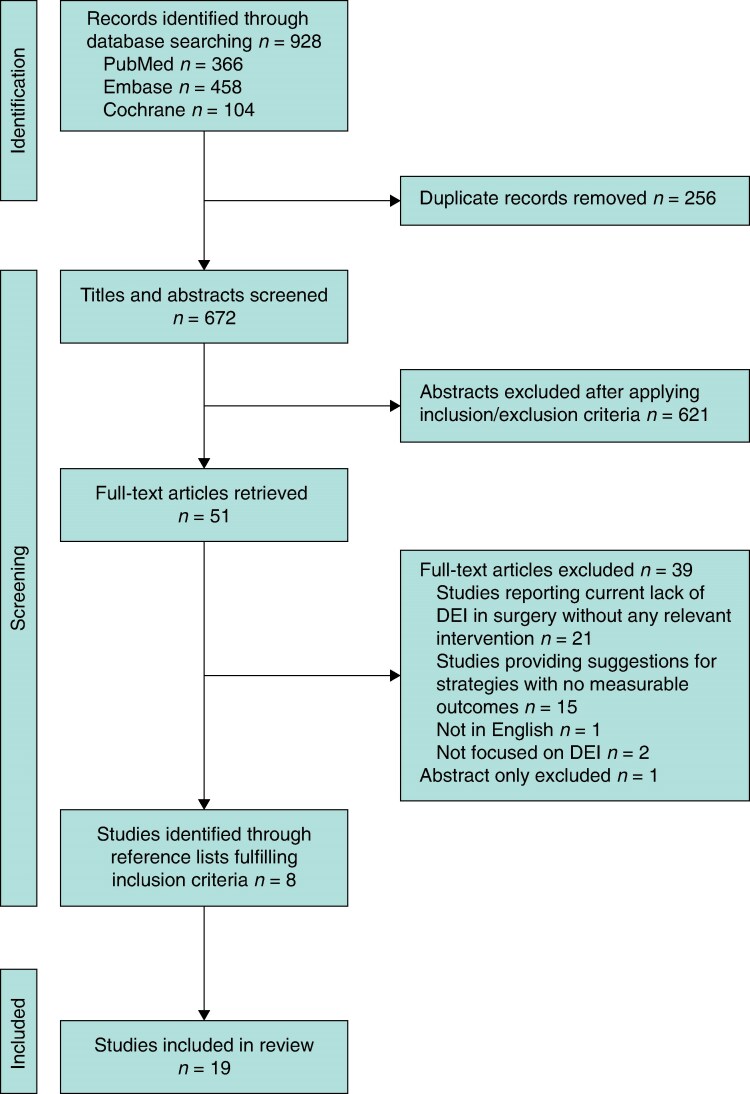
PRISMA-ScR diagram summarizing the literature review process DEI, diversity, equity, and inclusion.

**Fig. 2:**
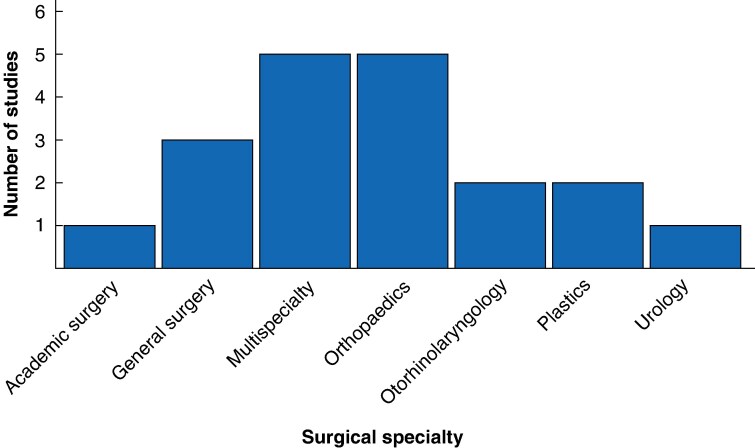
Distribution of studies across surgical specialties focusing on interventions that promote diversity, equity, and inclusion Multispecialty encompasses combination of different specialties including academic, general surgery, orthopaedics, otorhinolaryngology, plastics, urology, vascular, thoracic, and neurosurgery.

The initial search identified 928 articles. After screening of abstracts and removal of duplicates, 51 full texts were assessed. A total of 11 studies met inclusion criteria and citation tracking identified 8 further papers, yielding 19 papers for final analysis (*Fig. 1*). Of these, eight (42%) targeted both women and UiM, four (21%) focused on women, and seven (37%) on UiM. All included articles were published between 2010 and 2025, predominantly from the USA with one study from the UK, despite no country restrictions being applied to the search strategy. Distribution of studies across surgical specialties can be seen in *Fig. 2*. Four main themes of interventions were identified: (a) pipeline programmes, (b) early-phase recruitment strategies, (c) mentorship, and (d) comprehensive institutional efforts. An overview of the primary characteristics of these four main themes is included in *Tables 1–4*, respectively.

**Table 1:** Summary of pipeline programmes

Study and year	Surgical specialty	Intervention	Description	Outcome metrics	Findings
Earp *et al.* (2020)^22^	Orthopaedics	Workshop and lectures	Perry Initiative’s MSOP: 3-hour programme with lectures, workshops, and skills lab	Recruitment rates; change in interest toward orthopaedics among female MS	Alumnae recruited rose from 28% to 31%; 43% initially undecided or uninterested, 25% became interested after the intervention. Positive shifts in perceptions of orthopaedics (lifestyle, diversity, training, and so on; *P* < 0.050 for all). Intellectual interest increased (*P* = 0.001).
Lattanza *et al.* (2016)^38^	Orthopaedics	Clerkship	Mandatory 3rd-year musculoskeletal clerkship	Rates of women and UiM MS applicants	Significant increase in the percentage of 3rd-year students rotating to orthopaedics (25%; *P* < 0.0001). Significant increase in female (81%) and UiM (101%) applicants. Successful applications remained the same (6%).
Vajapey *et al.* (2019)^30^	OHNS	Clerkship and mentorship	Structured clerkships with mentorship	UiM clerkship participation, applications, publications	7 of 15 applied to OHNS training, 6 were appointed successfully. UiM representation in training grew from 9% to 16%. Students reported clerkship influenced their decision to pursue OHNS and increased interest in academic medicine.
Williams *et al.* (2021)^31^	Orthopaedics	Clerkship and mentorship	8-week immersion clerkship and mentorship	Female and UiM orthopaedics applications *versus* national controls	Women had increased odds (OR 51.3; *P* < 0.001) and UiM had increased odds (OR 14.5; *P* < 0.001) of applying to orthopaedics.
London *et al.* (2016)^21^	Multiple procedural specialties: general surgery; orthopaedics; anaesthesiology; neurosurgery; EM; urology; O&G	Clerkship and mentorship	8-week immersion clerkship and mentorship	Female and UiM application rates to procedure-based specialties	75% of students applied to procedure-based specialties; 72.3% entered different procedure-based specialties.
Nellis *et al.* (2016)^32^	Orthopaedics	Scholarship and mentorship	Scholarships for attending national meetings and mentorship	Female MS pursuing orthopaedics who received an award *versus* those who did not	80% of recipients pursued orthopaedics compared with 45% non-recipients; 55% pursued alternate career paths.
Mason *et al.* (2016)^39^	Orthopaedics	Workshop and lectures	B.O.N.E.S.: half-day skills (casting, suturing, and so on), panel discussions, and counselling	Interest and recruitment rates to specialty training among female MS	After the workshop, 55.5% showed increased interest; 37.3% recruited for training.
Mason *et al.* (2017)^40^	Thoracic and general surgery	Scholarship and mentorship	Women in thoracic surgery: scholarship and mentorship for women in an accredited thoracic/GS programme, and women with postdoctoral qualifications who completed medical school	Career progression in women awardees *versus* peers/non-awardees	Significant higher progression rates towards CTS; 71% of MS awardees entered specialty training; 26% in integrated CTS programmes *versus* < 0.1% of MS non-awardees (*P* < 0.0001); 37% in general surgery *versus* 4.8% of MS non-awardees, (*P* < 0.0001). Among GS trainee awardees, 59% pursued CTS fellowship *versus* 7.7% non-awardees (*P* < 0.001). 74% of CTS awardees completed training; 41% became academic surgery faculty members.

Clerkships were also discussed by Butler *et al*.^41^, Llado-Farrulla *et al*.^42^, Williams *et al.*^43^, and Lin *et al.*^44^, as seen in *Tables 2*, *4*. MSOP, Medical Student Outreach Programme; MS, medical student; UiM, under-represented in medicine; OHNS, otolaryngology head and neck surgery; OR, odds ratio; EM, emergency medicine; O&G, obstetrics and gynaecology; B.O.N.E.S., Bringing Orthopaedics to New England Students; CTS, cardiothoracic surgery; GS, general surgery.

**Table 2:** Summary of early-phase recruitment strategies

Study and year	Surgical specialty	Intervention	Description	Outcome metrics	Findings
Gardner *et al.* (2020)^45^	General surgery, orthopaedics, plastic surgery, urology, OHNS, vascular/thoracic surgery, neurosurgery	Clerkship, mentorship, selection tool, holistic review, diversity committee, faculty training	Three-faceted approach including clerkship, holistic reviews, and targeted outreach/mentorship	UiM interviewees and specialty placement rates	UiM representation in interviewees increased from 11.2 to 18.8% (*P* < 0.05). UiM specialty placement increased from 9.1 to 23.5%. No significant change in USMLE scores or ranking.
Butler *et al.* (2019)^41^	General surgery	Selection tool	Lowered USMLE cut-offs, inclusion of SJT	UiM and female invitations and interviews	Increased female (47.5%) and non-White (52.7%) applicants; increased UiM interviews (8%; *P* < 0.01).
Llado-Farrulla *et al.* (2021)^42^	General surgery	Selection tool, holistic review, diversity committee, faculty training	Flexible USMLE step 1 cut-off, implicit bias training, holistic review scoring	Ranking and specialty appointment among women and UiM applicants	Significant increase in ranked women (42→61%) and UiM (14→20%); appointment increased but not significantly (*P* = 0.11)
Williams *et al.* (2023)^43^	Plastic surgery	Clerkship, mentorship, holistic review	Multifaceted recruitment with clerkships and mentorship, holistic review, targeted outreach	UiM and female representation after intervention	UiM recruitment increased from 0% to 29%, whereas women remained at 27–29%.
Nehemiah *et al.* (2020)^46^	General surgery	Holistic review, diversity committee	Holistic review of specialty training applications	UiM invited, interviewed, ranked, and appointed	Significant increases in UiM invited (4% to 27%; *P* = 0.0007), interviewed (4% to 25%; *P* = 0.0007), and ranked (4% to 25%; *P* = 0.0036). Appointment rate increased to 30% (*P* = 0.06).
Thompson *et al.* (2022)^47^	Urology	Clerkship, mentorship, holistic review	Three-faceted intervention: funded clerkships and mentorship, holistic application review, and targeted outreach	UiM interviews and specialty entry rate	UiM interviewees increased from 6% to 40%; UiM entry rates rose from 0% to 25–50%; completed specialty training increased from 10% to 30–35%. No significant difference in USMLE scores or ranking.

*Dossett et al.* ^48^ discussed holistic review as part of their multifaceted approach, as seen in *Table 4*. OHNS, otolaryngology head and neck surgery; UiM, under-represented in medicine; USMLE, US Medical Licensing Exam; SJT, situational judgement test.

**Table 3:** Summary of mentorship dominant programmes

Study and year	Surgical specialty	Intervention	Description	Outcome metrics	Findings
Butler *et al.* (2010)^49^	General and academic surgery	Mentorship	Diverse Surgeons Initiative (skills sessions, surgical labs, ABSITE preparation, mentorship)	Fellowship uptake and academic appointments among UiM	86% pursued fellowships; 57% appointed as academic surgery faculty members.
Butler *et al.* (2015)^50^	General and academic surgery	Mentorship	Long-term follow-up of DSI outcomes (fellowships, academic positions, leadership)	Fellowships, academic roles, leadership, and research contributions	87% completed fellowships; 41% secured faculty positions; 18% held leadership roles; 76% contributed to research.
Rehman and Kungwengwe *et al.* (2025)^51^	Plastic surgery	Mentorship	INSPIRE pilot programme (mentorship with background-matched mentors)	Research engagement and output (presentations/publications), career interest	Majority engaged in research outputs (77% publications, 59% national presentations); interest in academic and plastic surgery increased by ∼40%.

Mentorship was paired alongside various other strategies in nine studies—Vajapey *et al.*^30^, Williams *et al.*^31^, Nellis *et al.*^32^, Mason *et al.*^39,40^, Butler *et al.*^41^, Llado-Farrulla *et al.*^42^, Williams *et al.*^43^, and Lin *et al.*^44^, as seen in *Tables 1, 2*, *4*. ABSITE, American Board of Surgery In-Training Examination, UiM, under-represented in medicine; DSI, Diverse Surgeons Initiative; INSPIRE, Inclusivity in Plastic Surgery—Insights, Recommendations, and Education.

**Table 4:** Summary of comprehensive institutional strategies

Study and year	Surgical specialty	Intervention	Description	Outcome metrics	Findings
Lin *et al.* (2016)^44^	Otolaryngology	Clerkship, mentorship, diversity training	Multifaceted intervention including forming diversity committee, salary parity audits, mentorship, clerkships	UiM and female faculty representation	Increased female and UiM faculty members; enhanced leadership roles for women and UiM. Salary parity achieved.
Dossett *et al.* (2019)^48^	Academic surgery	Holistic review, diversity committee, and faculty training	Diversity committee, implicit bias training, ‘Rooney Rule’, holistic recruitment	Representation of UiM and women in surgical training positions	Increased recruitment and hiring of women (50%) and UiM (33%).

Butler *et al.*^41^, Nehemiah *et al.*^46^, and Thompson *et al.*^47^ also used these strategies as seen in *Table 2*. UiM, under-represented in medicine.

### Pipeline programmes

Pipeline programmes to increase surgical interest and appointment rates among women and UiM students were a common strategy seen in 12 of 19 studies, through workshops, lectures, scholarships, and clerkships (*Table 1*).

Two orthopaedics studies^22,38^ used workshops and lectures to influence female medical students’ interest and specialty selection outcomes. The nationwide Perry Initiative’s Medical Student Outreach Programme^38^, led by female orthopaedic surgeons, boosted early interest and intellectual curiosity (*P* = 0.001), and nearly doubled alumnae orthopaedic surgery entry rates over a decade following a 3-hour lecture and a 90-minute hands-on workshop. The Bringing Orthopaedics to New England Students half-day workshop^22^ included participants across all years of medical school featuring skills stations, panel discussions, and counselling. After the workshop, 55.5% of attendees reported a new interest in orthopaedics, 39.3% planned to pursue it as a career, and 37.3% were appointed into orthopaedic training programmes^22^. Senior students had a significantly higher specialty selection rate (61%) compared with first- and second-year students (22%). Although this suggests differential attainment within the group, the variation likely reflects baseline differences in exposure and pre-existing interest, rather than the workshop alone. Without a control group of non-attendees, it is not possible to isolate a causal programme effect on specialty placement outcomes. However, the overall increase in reported interest across participants suggests that the workshop may still have had a general positive effect on engagement with the specialty.

Scholarships are stipends provided to cover expenses such as travel, housing, and/or fees for attending national surgical meetings^30,31^. In a 13-year follow-up national study^30^, 80% of female recipients entered orthopaedics versus 45% of female non-recipients, suggesting that scholarships may reinforce commitment. A separate study^31^ showed that funding medical students and doctors in training (DiT) to attend thoracic surgery conferences advanced their careers to faculty positions at significantly higher rates, suggesting that financial support at different transition points can lead to long-term engagement.

Clerkships, also referred to as *internships*, lasting 4–8 weeks offer clinical exposure, mentorship, and academic support^21,32,39,40^. A mandatory 1-month musculoskeletal clerkship for medical students led to an 101% increase in UiM applications and an 81% increase in women applying to orthopaedic training programmes^21^. Although limited to one institution, comparisons with national data and post-clerkship surveys supported its influence, suggesting potential generalizability. A small study (15 medical students) by Nellis *et al*.^32^ showed an 85% selection success in otorhinolaryngology, with UiM representation nearly doubling, but it lacked a comparator group. The three-phase Nth dimension programme, a non-profit pipeline initiative to increase diversity in orthopaedic surgery, combined early exposure, structured clerkships, and mentorship. This was associated with women and UiM participants having 51 and 15 times higher odds, respectively, of applying to orthopaedics compared with national controls, and a 76% orthopaedics placement rate^39^. However, the absence of phase-specific tracking and potential selection bias limits conclusions about the clerkship’s independent impact. Clerkships also feature in holistic reviews and broader institutional strategies^41–44^, discussed below.

### Early-phase recruitment initiatives

Traditionally, entrance exams played a key role in training selection. A multicentre general surgery study^45^ trialled a two-step process using situational judgement tests with lowered exam cut-offs to prioritize attributes like professionalism, resilience, and teamwork. This led to an 8% increase in interview offers to UiM applicants. However, without tracking final entry outcomes, it remains unclear whether this approach achieved true equity or compromised standards (*Table 2*).

The holistic selection approach balances academic achievements with personal attributes and experiences^52^. This comprehensive assessment was associated with increased interviews and appointments of women and UiM applicants without compromising academic standards^41–43,46,47^. For instance, programmes that lowered entrance exam cut-offs reported improved diversity while maintaining median in-training exam scores above national averages^46^. Some centres retained academic thresholds^47^ whereas others incorporated targeted outreach and funded clerkships^41–43^, reporting higher completion rates and zero attrition among appointed DiT across multiple hospitals and specialties on long-term follow-up^41–43^. Although it is difficult to isolate the impact of each component in the three-pronged approach, these studies support the value of structured, equity-focused recruitment for both short- and long-term outcomes.

### Mentorship opportunities

Mentorship featured prominently in twelve studies in this review^30–32,39–44,49–51^, with the national Diverse Surgeons Initiative (DSI) standing out for its support of UiM DiT through skills development, career guidance, and networking (*Table 3*). The DSI led to increased fellowship and faculty appointments, particularly in minimally invasive surgery, with 86% of its 64 graduates pursuing fellowships, exceeding national averages^49,50^, and over half securing full-time academic faculty roles. Outcomes included high retention and leadership attainment over five years, suggesting lasting impact. More recently, the Inclusivity in Plastic Surgery—Insights, Recommendations, and Education pilot programme demonstrated the value of identity–concordant mentorship, with 77% of participants producing research outputs and ∼40% reporting increased interest in academic and plastic surgery careers. However, the scarcity of UiM DiT in such initiatives often precluded control comparisons and the potential selection bias limited definitive conclusions. Mentorship was also integrated within scholarships, clerkships, and holistic recruitment efforts^30–32,39–44^, though its independent impact was often not evaluated. Nonetheless, participants consistently reported mentorship to have positive influences on career decision-making and progression.

### Comprehensive institutional culture

Sustainable DEI efforts often involved institutional policies and leadership engagement. Diversity committees^41,46,47^, dedicated diversity directors^44^, broad job promotion, holistic interviews, and rule-based interventions (for example, the modified Rooney Rule, which mandated selecting two diverse candidates for each position) were used to ensure fair recruitment across all levels^48^ (*Table 4*). These efforts increased short-term representation and longitudinal review showed attainment of salary parity among faculties. Faculty training was also key, including implicit bias workshops, diversity retreats, speaker events, book clubs, and an endowed lectureship recognizing women leaders^41,44,46,48^. Although widely adopted, the multifaceted nature of these interventions makes it difficult to isolate their individual impact.

## Discussion

This review demonstrates that DEI efforts have improved representation of women and UiM individuals in surgery, but widespread implementation remains limited and broader demographic shifts have been modest^18^. To improve uptake, DEI interventions must be contextualized within the structural and cultural realities of surgical practice. The heterogenous nature of DEI programmes posed challenges in isolating the specific impact of individual elements. Multimodal approaches were most prevalent (12 of 19 studies), with mentorship the most frequently integrated component (63%). Longitudinal mentoring was consistently associated with improved career progression, whereas structured sponsorship— where mentors actively advocate for their mentees by providing access to high-impact opportunities and professional endorsements^31,53^—was less common but appears critical for promotion and leadership development. Future work should prioritize formalized sponsorship structures and address the ‘decanal divide’^54^, a phenomenon in which women and UiM individuals are under-represented in strategic and research leadership roles, often confined to mentoring and education-focused positions.

Despite promising strategies, outcome measures were often limited to short-term metrics such as application numbers, interview offers, or hiring rates. Although helpful, these do not reliably predict long-term impact. Some studies employed structured interviews and bias training to improve recruitment fairness, but inter-rater subjectivity and implicit bias persisted. Incorporating diverse selection panels and regular bias mitigation training may help address these limitations^55,56^. Longer-term outcomes, such as retention, faculty promotion, and leadership appointments, were reported less frequently, typically through single intervention with extended follow-up^43,47^, multicareer stage strategies^31^, or multifaceted approaches^44,48–50^. Programmes featuring longitudinal mentorship, leadership support, consistent evaluation, and institutional accountability showed better outcomes over time. These more meaningful metrics should be prioritized in future evaluations.

Representation itself emerged as a key driver^22,30,31,38,41–47,52^, and students were more likely to pursue surgical specialties when they saw mentors or leaders who reflected their background, aligning with the attraction–selection–attrition model^57^. Notably, cross-gender mentorship showed comparable success to same-gender mentoring^58^, suggesting there can be flexibility in addressing the mentorship gap.

A key challenge in DEI implementation is identifying where to focus resources and how scalable interventions are. This review shows that certain strategies like holistic reviews and mentorship have been effectively applied across different training levels and specialties^36,41^. Given shared features across surgical disciplines, such as hierarchical structures and high-pressure environments^59^, such strategies could theoretically translate across grades and specialties. However, the generalizability of findings across different countries remains limited, as the majority of the included studies originated from the USA, with only one recent UK-based initiative identified. Despite an inclusive search strategy, evidence from other healthcare systems remains scarce. This may reflect differences in research focus rather than a lack of awareness or interest internationally. For example, studies from the UK^60^ and Europe^61^ continue to show disparities in surgical workforce demographics, yet formal evaluations of DEI interventions are comparatively rare. Differences in healthcare system structures may influence institutional priorities. In the USA, where care is largely privately financed and delivered, DEI outcomes may be more closely tied to institutional reputation and financial incentives^62^. In contrast, systems such as those in the UK, Canada, and Europe^63^, with varying degrees of public and private involvement, may prioritize service delivery and equitable access, with different incentives for evaluating and implementing DEI initiatives. Additionally, the historical context of race and gender^64^ in the USA may make DEI a more politicized and publicly scrutinized issue. The predominance of US-based literature could also reflect publication bias, underscoring the need for more robust evaluation and reporting of DEI efforts from other healthcare systems to inform global applicability. The absence of such studies likely reflects a gap in testing and reporting solutions rather than in problem recognition.

Health disparities contribute to significant healthcare costs, making DEI initiatives a major resource consideration^65^. Feasibility and sustainability considerations, though infrequently addressed in the papers, remain relevant. A few studies discussed funding, primarily for pipeline initiatives like workshops^22^, clerkships, and scholarships^30,41–43^, and showed positive results in increasing applicants from minority groups but were limited in scale and sustainability. More sustainable, low-resource approaches like holistic review delivered notable returns by diversifying the pipeline without sacrificing academic standards. Resource-intensive efforts like hiring DEI leads or running institutional training programmes offered longer-term institutional benefits but remained limited by financial capacity. Additionally, the burden of DEI work often falls disproportionately on staff from minority groups, a phenomenon known as the ‘minority tax’^7,48,66^. Though difficult to quantify, this invisible workload can hinder professional advancement and contribute to higher attrition rates. Some studies addressed this by additionally encouraging involvement of faculty who were not from minority groups to take active role in DEI efforts^49,50^, helping to distribute responsibilities more equitably and promote institutional accountability.

An intrinsic limitation of literature-based reviews is their reliance on the methodological design, quality, and rigour of the included studies. Most studies reviewed here were retrospective, which may have introduced recall bias and uncontrolled confounding variables. Additionally, important information may have been missed due to incomplete study details, constraints related to search terms, or inaccessible materials. Most studies were from the USA, limiting generalizability to other healthcare systems. The heterogeneity of interventions made it difficult to isolate the most effective components. Although efforts were made to conduct a comprehensive review, these limitations should be considered when interpreting the findings.

Future research should focus on longitudinal and randomized designs with control groups to strengthen causality. Standardized outcome measures should be developed to evaluate recruitment, retention, and leadership progression. Expanding DEI efforts beyond the USA is crucial to understanding their impact in different healthcare models, such as publicly funded systems. Research should also include diverse groups, such as individuals with disabilities, lower socioeconomic backgrounds, and varying gender identities. Efforts to offset the minority tax by recognizing and incentivising DEI contributions are also essential. Refining mentorship programmes to include structured sponsorship and interdisciplinary models could enhance scalability. By adopting these recommendations, the surgical field can move towards more diverse, inclusive, and equitable practices, improving patient outcomes and strengthening patient–physician relationships.

The conversation around DEI in surgery has shifted from identifying issues to actively implementing and evaluating solutions, especially over the last decade. A growing body of research outlines targeted strategies and their outcomes. This review highlights that effective DEI interventions in surgery focus on pipeline programmes, holistic recruitment, mentorship, and institutional strategies. Workshops, scholarships, and clerkships have increased interest and applications from women and UiM individuals by addressing negative perceptions and offering early surgical exposure. Holistic reviews in specialty training applications have improved diversity without lowering standards, shifting from rigid examinations to broader evaluations. Long-term mentorship has supported career progression, and diversity committees, bias training, and salary parity have fostered more inclusive environments, improving retention. Overall, mentorship, holistic reviews, scholarships, and institutional strategies have effectively enhanced recruitment and career advancement while being resource efficient, guiding where leaders should focus resources. However, sustainable implementation and broader applicability across healthcare systems remain key challenges. Future efforts should refine these interventions to ensure long-term impact and alignment with systemic changes in surgical education and practice.

## Supplementary Material

zrag020_Supplementary_Data

## Data Availability

This study is based on previously published data. All articles included in the review are available to researchers through the public databases PubMed, Embase, and the Cochrane Library. The list of included studies and full search strategy are provided in the manuscript and *Supplementary material*.
