# Supplementary material for: Exploring effective strategies in promoting diversity, equity, and inclusivity in surgery: scoping review
Source: BJS Open. 2026 Apr 13;10(2):zrag020. doi: 10.1093/bjsopen/zrag020 (PMC13075988; doi:10.1093/bjsopen/zrag020)
Supplement: zrag020_Supplementary_Data [file zrag020_supplementary_data.docx]

**Exploring effective strategies in promoting diversity, equity and inclusivity (DEI) in Surgery – scoping review**

Afroza Sharmin^1^, Rawan Ahmed^2^, Alexander W Phillips^3,4^

^1^Department of Surgery and Cancer, Imperial College London, London, UK

^2^Bioscience Education Department, King’s College London, London, UK

^3^Northern Oesophagogastric Unit, Royal Victoria Infirmary, Newcastle upon Tyne, UK.

^4^School of Medical Education, Newcastle University, Newcastle Upon Tyne, UK.

***Correspondence to*** – Dr. Afroza Sharmin, Department of Surgery and Cancer, Hammersmith Hospital, Du Cane Road, London, W12 0HS

Email – [a.sharmin@ic.ac.uk](mailto:a.sharmin@ic.ac.uk)

sharmin.afroza@nhs.net

**Orcid ID** - 0000-0003-3038-5506

**Supplementary Materials - Index**

| **Supplementary Methods** |  |
| --- | --- |
| A. Search strategy | *Page. 2* |
| B. Adapted Critical Appraisal Skills Programme (CASP) checklist | *Page. 3* |

**Supplementary Methods**

**A. Search strategy**

Search terms were kept broad to capture maximum studies in this research area. The electronic databases (PubMed, Embase and the Cochrane Library) were searched using the following combinations to retrieve pertinent results for the study –

- Surg* AND (Diversity OR Equity OR Inclusivity) AND (Strateg* OR Initiative)

- Surg* AND (Equity OR Divers* OR Inclus*) AND (Strateg* OR Initiative)

- Surg* AND (Equity OR Divers* OR Inclus* OR DEI OR EDI OR Underrepresented) AND (Strateg* OR Initiative)

- Surg* AND (Diversity AND Equity AND Inclusivity) AND (Strateg* OR Initiative)

- Surg* AND EDI AND (Strateg* OR Initiative)

- Surg* AND DEI AND (Strateg* OR Initiative)

- Surg* AND Underrepresented AND (Strateg* OR Initiative)

**B. Adapted Critical Appraisal Skills Programme (CASP) checklist**

| **Title of the study** | | | | |
| --- | --- | --- | --- | --- |
| **Question** | **Yes** | **Can’t tell** | **No** | **Comments** |
| **Section A- Are the results of the study valid?** | | | | |
| 1. Did the study address a clearly focused issue related to DEI in surgery? |  |  |  |  |
| 2. Was the appropriate cohort selected as per the inclusion criteria? |  |  |  |  |
| 3. Were the strategies for promoting DEI accurately described? |  |  |  |  |
| 4. Were the outcomes of DEI strategies relevant and accurately measured to minimize bias? |  |  |  |  |
| 5. (a) Have the authors identified all-important confounding factors that could affect DEI outcomes?  5. (b) Have they accounted for these confounding factors in the design and/or analysis of the review? |  |  |  |  |
| 6. (a) Was the follow up of subjects complete enough to ensure comprehensive results?  6. (b) Was the follow-up period of included studies long enough to observe significant DEI outcomes? |  |  |  |  |
| **Section B - What are the results?** | | | | |
| 7. What are the key findings of this review regarding effective DEI strategies in surgery? |  |  |  |  |
| 8. How precise are the findings (e.g. confidence intervals, statistical significance)? |  |  |  |  |
| 9. Do you believe the results based on the evidence provided? |  |  |  |  |
| **Section C- Will the results help locally?** | | | | |
| 10. Can the results be applied to the local surgical population? |  |  |  |  |
| 11. Do the findings of this review align with other available evidence on DEI strategies in healthcare? |  |  |  |  |
| 12. What are the practical implications of this review for promoting DEI in surgical practice? |  |  |  |  |

**Supplementary Table 1 - Adapted Critical Appraisal Skills Programme (CASP) checklist**
